# Different Trends of Immune Activation Markers When Switching to Either Oral or Injectable Dual Antiretroviral Therapy Based on Integrase Inhibitors in People Living with HIV

**DOI:** 10.3390/pathogens15030316

**Published:** 2026-03-14

**Authors:** Matteo Vassallo, Jacques Durant, Roxane Fabre, Jacqueline Capeau, Soraya Fellahi, Jean-Philippe Bastard, Pierre Corbeau, Christian Pradier

**Affiliations:** 1 Department of Internal Medicine/Infectious Diseases, Cannes General Hospital, 06400 Cannes, France; 2 Unité de Recherche Clinique Cote d’Azur (Unité de Recherche Radiologically Isolated Syndrome), Université Côte d’Azur, 06000 Nice, France; 3 Department of Infectious Diseases, Archet Hospital, Nice University, 06202 Nice, France; jacques.durant@free.fr; 4 Public Health Department, Archet Hospital, Nice University, 06202 Nice, France; fabre.r@chu-nice.fr (R.F.); pradier.c@chu-nice.fr (C.P.); 5 Pain Department and FHU InovPain, Nice University Hospital, Cote Azur University, 06000 Nice, France; 6 Institut Hospitalo-Universitaire de Cardiométabolisme et Nutrition (ICAN), RHU CARMMA, Centre de Recherche Saint-Antoine, INSERM UMR_S 938, Sorbonne Université, 75012 Paris, France; jacqueline.capeau@inserm.fr (J.C.); soraya.fellahi@aphp.fr (S.F.); 7 Département de Biochimie-Pharmacologie, Assistance Publique-Hôpitaux de Paris, Hôpitaux Universitaires Henri Mondor, 94000 Créteil, France; jean-philippe.bastard@aphp.fr; 8 FHU-SENEC, INSERM U955 and Faculté de Santé, Université Paris Est (UPEC), UMR U955, 94010 Créteil, France; 9 Laboratory of Immunology, Nimes University Hospital, 30029 Nimes, France; pierre.corbeau@igh.cnrs.fr

**Keywords:** HIV, simplification strategies, dual drug regimen, immune activation

## Abstract

Background: Despite improvements in life expectancy, people living with HIV (PWH) continue displaying immune activation and high rates of comorbid conditions. No comparative studies concerning activation markers exist between simplification strategies to either oral or long-acting (LA) dual ART. Methods: We prospectively collected plasma samples from PWH on successful ART, simplifying treatment from triple oral to either oral or LA dual ART based on integrase inhibitors. We measured changes in soluble CD14 (sCD14), soluble CD163 (sCD163), monocyte chemoattractant protein-1, and interleukin-6. Background measurements and markers of microbial translocation and gut integrity (I-FABP, LBP) were also collected. Results: From 2019 to 2023, 38 PWH were analyzed (mean age 52, 87% male, 21 years HIV diagnosis, CD4 730 cells/mm^3^, nadir CD4 317 cells/mm^3^, AIDS 13%). After 7.2 months, sCD14 trajectories differed according to regimen (+0.43 ng/mL, *p* = 0.033 for LA ART, −0.62 ng/mL, *p* < 0.001 for oral ART) but were not related to I-FABP or to LBP values. In case of CD4 nadir < 200 cc/mm^3^, AIDS, or very-low-level viremia, sCD163 values significantly increased when switching to oral but not to LA dual ART. Conclusion: We found different trends in immune activation markers and risk factors associated with PWH switching to either oral or LA ART, requiring larger studies.

## 1. Introduction

The availability of antiretroviral treatment (ART) led to a dramatic improvement in life expectancy among people living with HIV (PWH), converting a severe and often fatal disease into a chronic condition [1,2,3]. However, the prevalence of non-AIDS-defining complications is still higher in PWH than in uninfected subjects [4]. The current leading hypothesis is that HIV infection is associated with a chronic inflammatory condition, in which there is increased activation of immune cells, associated with several non-AIDS-defining comorbid conditions [5,6,7]. The causes of such immune activation are still incompletely understood. Possible explanations include persistent low-level HIV replication, microbial translocation, and co-infections [8,9,10,11,12,13].

Since the introduction of simplification strategies with dual-drug ART, most studies report similar efficacy on plasma virological control when compared to triple-drug therapies. However, some questions have been raised regarding the potential consequences on immune activation of reducing the number of molecules, and data are contradictory. Indeed, the TANGO and SWORD studies did not show any significant change in immune activation markers [14,15], but we and others previously reported that simplification to oral dual-drug ART could be associated with increased monocyte–macrophage activation, a well-known marker of chronic inflammation, particularly in subjects with a low CD4 nadir or a previous AIDS-defining condition [16,17,18].

The intramuscular Cabotegravir and Rilpivirine combination is the first complete long-acting (LA) dual regimen introduced among the guidelines for the maintenance of HIV-1 virologic suppression [19,20], and studies have shown its non-inferiority in terms of treatment success [21,22]. However, information on immune activation when switching to injectable dual ART is still scarcer than for oral regimens [23,24,25], and no comparative data exist between oral and LA dual ART.

Our aim was to measure trends in immune activation markers, microbial translocation, and associated risk factors in patients simplifying their treatment from triple oral ART to either an oral or an LA dual regimen.

## 2. Materials and Methods

### 2.1. Study Design and Participants

Patients who participated in either the Immunadapt or the Longadapt study were included in this analysis.

Immunadapt is a single-arm prospective study exploring immune activation markers in successfully treated subjects on stable triple ART, switching to an oral dual ART.

Longadapt is a single-arm prospective study aiming to assess the impact on immune activation markers of switching from a triple-drug regimen to LA Cabotegravir and Rilpivirine in PWH on stable ART.

For both studies, patients were selected among those followed in the Department of Internal Medicine in Cannes General Hospital and the Infectious Diseases Department in Nice University Hospital, France. Each individual was routinely followed in our departments, and meeting the inclusion criteria was required to participate. Recruitment continued until 20 patients were included, which was the sample size estimated necessary for the analysis in both studies.

The following inclusion criteria were applied: HIV-1-infected subjects on stable and successful ART (viral load < 50 copies/mL for at least 6 months, measured with Xpert© viral load, Cepheid AB, France or Aptima™ HIV Quant Dx, Hologic, France), switching from an oral triple-drug regimen to an oral dual-drug regimen for Immunadapt, and switching to an injectable Cabotegravir and Rilpivirine combination for Longadapt. In the Longadapt study, subjects undergoing the 28-day lead-in phase with oral drugs preceding parenteral administration were also included.

Patients who were not on stable and successful ART, or those whose treatment included a different number of compounds, such as those switching from a quadruple to a triple-drug regimen, were excluded.

The Immunadapt study was approved by the Paris Ethics Committee (Comité de Protection des Personnes, Ile de France IV, ID-RCB 2019-A00159-48), and the Longadapt study was approved by the Necker Ethics Committee (Comité de Protection des Personnes, Ile de France II, Hopital Universitaire de Necker Enfants Malades Carré Necker, Paris, ID-RCB 2022-A00280-43). For both studies, patients gave written informed consent to participate.

### 2.2. Plasma Markers of Immune Activation and Microbial Translocation

Measurement of immune activation was performed just before the ART switch and at least 6 months later. In case of viral replication >50 copies/mL during follow-up, patients were excluded from the analysis. For the Immunadapt analysis, markers were measured in the Laboratory of Immunology in Montpellier Hospital, France, while for the Longadapt study analysis, they were performed in the Laboratory of Biochemistry and Pharmacology in Henri Mondor Hospital, Creteil, France.

In both studies, the following markers were measured: IL-6 (ProcartaPlex ImmunoAssays, Life Technologies SAS for Immunadapt, Roche, Cobas-8000 for Longadapt), monocyte chemoattractant protein-1 (MCP-1) (ProcartaPlex ImmunoAssays, Life Technologies SAS for Immunadapt, Quantikine ELISA, Biotechne SA for Longadapt), sCD14, and sCD163 (Quantikine ELISA, Biotechne SA for both studies). These latter assays were performed at different times with different batches, using different antibodies, which allows for comparison of the values within each study but precludes direct comparison between the two studies.

Microbial translocation was evaluated by measuring lipopolysaccharide-binding protein (LBP) with Quantikine ELISA kits for both studies.

Moreover, intestinal fatty acid binding protein (I-FABP) (Quantikine ELISA BiotechneSA), a marker of gut integrity [26], was measured in the Longadapt study.

### 2.3. Demographic Parameters and Background Measurements

Demographic and main viro-immunological characteristics were recorded.

During follow-up, plasma viral load was quantified between 2 and 3 months after switching treatment. Viro-immunological parameters were measured at the end of follow-up, i.e., at least 6 months after treatment simplification, together with immune activation markers.

Viral blips were defined as transient low-level viremia (LLV), between 50 and 500 copies/mL, preceded and followed by suppression, i.e., below the quantification limit of the assay [27]. To assess the impact on immune activation, we also checked for very-low-level viremia (VLLV) detected at inclusion or during follow-up. VLLV was defined as viremia < 50 copies/mL detected by clinical assays with quantification cut-offs of <50 copies/mL [28].

During follow-up, clinical events and newly prescribed co-medications were also recorded.

### 2.4. Statistical Analysis

The main demographic and viro-immunological characteristics of the population were described. Using R-4.3.0 software, data from the Immunadapt and the Longadapt studies were compared using the Mann–Whitney–Wilcoxon test for quantitative parameters, while either Khi2 or Fisher’s exact tests were used for qualitative variables. Parameters associated with any significant change in immune activation markers during the follow-up were included in the multivariate model. Moreover, in case of significant variations of immune activation markers, factors associated with their trajectories were analyzed using Spearman’s correlation test for quantitative variables and the Wilcoxon–Mann–Whitney test for qualitative parameters. Finally, comparisons according to group risks for immune activation were performed using the Wilcoxon–Mann–Whitney test. According to previous publications, subjects at high risk for immune activation were defined by at least one of the following parameters: nadir CD4 < 200 cc/mm^3^, previous AIDS, or VLLV during the follow-up [14,29].

As the immune activation parameters in the two studies were not measured in the same laboratory, comparisons between markers were limited to the trajectories from the inclusion to the end of follow-up.

## 3. Results

### 3.1. Population Characteristics

Inclusions were performed between April 2019 and May 2020 for the Immunadapt study and from July 2022 to February 2023 for the Longadapt study. The main reasons for switching ART included reducing the number of pills, limiting potential long-term side effects of antiretrovirals, or improving the quality of life. Forty individuals were initially included. However, two subjects in the Longadapt study were excluded: in one case, for not respecting the inclusion criteria, and in another case, due to pregnancy. Therefore, 38 subjects were analyzed (mean age 54 years, 87% men, years since known HIV infection 21, number of ART regimens received before the switch 5, CD4/CD8 ratio 1, nadir CD14 318 cells/mm^3^; Table 1).

Demographic and background characteristics of PWH were similar in the two studies, except for a higher prevalence of subjects with hypertension and a trend of a larger number of ART regimens received in the Longadapt study (Table 1). Main triple ART before the simplification was similar in the two studies and in the large majority of cases included two nucleoside reverse transcriptase inhibitors (NRTI), associated either with an integrase strand transfer inhibitor (INSTI) or a non-nucleoside reverse transcriptase inhibitor (NNRTI) (Table 1). In the Immunadapt study, the main dual ART prescribed at the switch consisted of Dolutegravir and Rilpivirine (14/20 subjects, 70%), while in six cases (30%), therapy included Dolutegravir and Lamivudine, and all subjects in the Longadapt study received LA Cabotegravir and Rilpivirine.

### 3.2. Follow-Up and Trends in Immune Activation Markers

After 6.9 and 7.5 months of follow-up in the Immunadapt and Longadapt studies, respectively, all subjects maintained a viral load below 50 cp/mL. However, in the Immunadapt study, three individuals had VLLV at the inclusion and seven at the end of the follow-up, while in the Longadapt study, none had VLLV at the inclusion and just one at the end of follow-up.

No significant co-medications potentially interfering with immune activation markers were prescribed during follow-up for both studies. In the Immunadapt study, one subject suffered from ischemic stroke and one from pulmonary embolism just before the end of follow-up, while no clinical event was recorded in the Longadapt study.

At the end of follow-up, in the Longadapt study, we observed a statistically significant increase in sCD14 (+0.43 ng/mL, *p* = 0.033), while the other immune activation markers did not significantly change (Table 2). Moreover, even when excluding outliers for sCD163 and IL-6, the results did not change (Table S1).

In contrast, in the Immunadapt study, we found a significant decrease in sCD14 values (−0.62 ng/mL, *p* < 0.001), while a trend for an increase in sCD163 was observed (Table 2). The type of triple ART before the switch did not affect the trend of immune activation markers.

Additionally, neither sCD14 nor sCD163 was associated with lipid profile changes in both studies, and, in the case of switching to the LA regimen, the lead-in oral phase did not have any bearing on the course of immune activation markers. Moreover, the LBP trajectory did not differ in both studies (mean increase 7.6%, SD 48.2, *p* = 0.978 in the Longadapt study, 7.5%, SD 58.0, *p* = 0.622 in the Immunadapt study), while i-FABP mean values, available only in the Longadapt study, significantly decreased from the inclusion to the end of follow-up (1603 vs. 1222 pg/mL, *p* =0.043). However, i-FABP values did not correlate with immune activation markers, but they were positively associated with HDL cholesterol and negatively associated with triglyceride trends (Table S2).

Finally, as sCD14 and sCD163 are both markers of monocyte–macrophage activation, we measured their correlations in the Longadapt and Immunadapt studies, and we found that their trajectories were correlated only when switching to LA ART (Table S3). Figure S1 shows the trajectory of immune activation markers for each patient in both studies.

### 3.3. Risk Factors Associated with the Trajectory of sCD14

As the main result was a different trajectory of sCD14 between subjects switching either to injectable or to oral dual ART, we sought to analyze factors associated with sCD14 in order to exclude any potential bias. Longer HIV duration, switching to a dual therapy containing an NNRTI compound, and switching to an injectable dual ART were associated with an increase in sCD14 values, but in multivariate analysis, only HIV duration and the type of ART at the switch were predictive factors associated with the trajectory of sCD14 (Table 3). Such results were confirmed by the analysis of sCD14 percent changes, which were associated with the type of ART (Table 3).

### 3.4. Differences Between Subjects with Low and High Risk for Immune Activation

At the end of follow-up, comparison between subjects with high risks for immune activation, defined by low nadir CD4, previous AIDS or VLLV, and the others, showed different trends in sCD163 values between switching to oral (+25.49% vs. +0.53%, *p* = 0.031) and LA dual ART (+27.18% vs. 26.76%, *p* = 0.750, Table 4). Even when excluding the sCD163 and IL-6 outliers, the results did not change (Table S4). No difference was found according to the type of triple ART before the switch.

## 4. Discussion

In a population of PWH on stable and successful triple ART, we found different trends of monocyte–macrophage markers in the case of switching to either oral or to LA dual ART, despite similar characteristics at inclusion.

In particular, changes in sCD14 showed different trajectories, with a decrease in those switching to oral dual ART and an increase in those starting LA dual ART.

Moreover, PWH with a high risk of immune activation, defined by low nadir CD4, previous AIDS, or VLLV during the follow-up, had different trends of sCD163, according to the dual ART regimen, with those switching to oral ART but not those to LA showing a significant increase in such a marker.

These cytokines have distinct though overlapping associations with HIV and chronic inflammation. Indeed, sCD14 is generally shed by activated monocytes and macrophages in response to lipopolysaccharide produced by certain gut bacterial species and is considered to reflect gut immune activation [30]. However, it could also be produced by other cell types, such as neutrophils and enterocytes [30], and it has been reported as a strong predictor of mortality in the setting of treated PWH [31]. Our results are in line with previous studies showing that values decrease after switching to oral INSTI, suggesting a positive effect on immune activation of this class of antiretrovirals, although the underlying mechanisms have not been identified [32,33,34,35].

Reasons for increased levels of sCD14 after switching to LA ART, which were independent from the virological control in plasma, remain unexplained. One hypothesis could be that injectable INSTI achieves lower concentrations in the gut than oral INSTI, which has been previously associated with partial gut permeability restoration and a healthier microbiota [36,37,38]. Indeed, the main prescribed oral ART consisted of Dolutegravir and Rilpivirine, while subjects switching to LA received very similar compounds, suggesting that the route of administration could have an impact on the trajectory of immune activation markers. However, although we did not measure drug concentrations in the reservoir and intestinal microbiota, we found that the significant decrease in I-FABP levels was independent of immune activation and probably associated with changes in the lipid profile.

Other possibilities may include poorer penetration in lymph nodes, which represent one of the largest reservoirs, and different effects on integrated virions. Indeed, HIV infection has been associated with persistent inflammation within lymphoid tissue despite successful treatment [5,39], and INSTI penetration in lymph nodes is generally poor, although data are limited to oral compounds [4,40]. Moreover, ART drug injection instead of gut absorption could result in a lower drug concentration in gut-associated lymphoid tissue (GALT), which represents the major lymphoid organ fighting HIV infection, thus potentially increasing immune activation and inflammation in this reservoir.

Additionally, we found different trends of sCD163 according to the dual ART regimen chosen among subjects with a high risk for immune activation. Reasons for such differences are unexplained and require further prospective studies. We suggest that different drug penetrations in lymph nodes and GALT between oral and LA INSTI could have an impact on inflammation. Indeed, we found a higher incidence of VLLV after switching to oral than to LA dual ART, suggesting that diminishing the drug pressure in the reservoir could drive increased signals of immune activation, as previously shown in B-cell follicle, adipose tissue, or cerebrospinal fluid [41,42,43]. Moreover, Richter al. recently showed that a 20% change in successful ART has detectable values of ultra-low level p24, associated with markers of immune activation and exhaustion [44].

This study has several limitations due to its small size, the lack of HIV-DNA measurements, and the lack of pharmacological data on drug penetration in tissues, which requires further investigation of treatment-related inflammation outcomes. Moreover, it would have been interesting to include a control-matched group continuing therapy without any modification in order to exclude that cytokine changes may reflect the common effect of ART over time rather than the simplification of therapy. Several patients were taking chronic treatments for comorbid conditions at inclusion (treatment for hypertension, diabetes, and dyslipidemia), which could, obviously, impact their immune activation and inflammation profiles. However, these therapies had been taken chronically at enrolment and were not modified during follow-up. Therefore, they may not have interfered with the cytokine trajectories after enrollment.

Additionally, the immune activation assays used different batches and different antibodies and were performed at different times, which precluded standardization. To reduce the risk of bias, we limited the comparisons to changes in absolute count and percent values between the inclusion and the end of follow-up, and for each patient, the two-point measurements were performed at the same time and under the same conditions.

## 5. Conclusions

We observed different trends in immune activation markers and associated risk factors in PWH switching to either oral or LA ART. We suggest that the ART administration route could impact the level of residual immune activation, which could have potential consequences and requires larger studies.

## Figures and Tables

**Table 1:** Patient characteristics at baseline.

		All Subjects		Study				
				Longadapt (*n* = 18)		Immunadapt (*n* = 20)		
	*n*	Mean	[SD]	Mean	[SD]	Mean	[SD]	*p*-Value *
Age (years)	38	54.1	[9.2]	52.3	[10.1]	55.7	[8.2]	0.327
Years of HIV infection	37	20.6	[10.4]	17.6	[12.8]	23,2	[7.1]	0.166
CD4 nadir (cells/mm^3^)	34	316.9	[207.5]	306.0	[260.9]	325,5	[160.8]	0.275
Viral load zenith (copies/mL)	22	211,650.0	[482,457.9]	266,953.8	[618,850.3]	131,766.7	[156,196.1]	1.000
CD4 at the inclusion (cells/mm^3^)	38	729.7	[314.8]	800.7	[336.6]	665.8	[287.3]	0.279
CD8 at the inclusion (cells/mm^3^)	38	908.8	[453.6]	1068.0	[522.9]	765.5	[332.4]	0.063
CD4/CD8 ratio	38	1.0	[0.5]	1.0	[0.6]	0.9	[0.4]	0.872
ART regimens (*n*)	36	5.5	[3.9]	4.8	[4.6]	6.2	[3.2]	0.080
Months on current ART	38	52.8	[34.8]	49.6	[27.8]	55.7	[40.6]	0.715
Years on ART	37	16.5	[8.4]	14.6	[10.3]	18.4	[5.7]	0.206
	*n*	*n*	(%)	*n*	(%)	*n*	(%)	*p*-value **
Sex	38							0.653
Female		5	(13.2)	3	(16.7)	2	(10.0)	
Male		33	(86.8)	15	(83.3)	18	(90.0)	
Hypertension	38							0.039
No		23	(60.5)	14	(77.8)	9	(45.0)	
Yes		15	(39.5)	4	(22.2)	11	(55.0)	
Diabetes	38							1.000
No		34	(89.5)	16	(88.9)	18	(90.0)	
Yes		4	(10.5)	2	(11.1)	2	(10.0)	
Previous hepatitis C	36							0.231
No		33	(91.7)	17	(100.0)	16	(84.2)	
Yes		3	(8.3)	0	(0.0)	3	(15.8)	
Dyslipidemia	38							0.880
No		27	(71.1)	13	(72.2)	14	(70.0)	
Yes		11	(28.9)	5	(27.8)	6	(30.0)	
Chronic alcoholism	38							-
No		38	(100.0)	18	(100.0)	20	(100.0)	
Yes		0	(0.0)	0	(0.0)	0	(0.0)	
Psychoactive therapy	38							1.000
No		35	(92.1)	17	(94.4)	18	(90.0)	
Yes		3	(7.9)	1	(5.6)	2	(10.0)	
Previous AIDS	38							1.000
No		33	(86.8)	16	(88.9)	17	(85.0)	
Yes		5	(13.2)	2	(11.1)	3	(15.0)	
Oral ART before switch	38							0.214
2 NRTI + 1 INSTI		25	(65.8)	14	(77.8)	11	(55.0)	
2 NRTI + 1 NNRTI		11	(28.9)	4	(22.2)	7	(35.0)	
Other		2	(5.3)	0	(0.0)	2	(10.0)	
VLLV at inclusion	38							0.606
No		34	(89.5)	17	(94.4)	17	(85.0)	
Yes		4	(10.5)	1	(5.6)	3	(15.0)	
Nadir CD4	34							0.417
≤200 cells/mm^3^		8	(23.5)	5	(33.3)	3	(15.8)	
>200 cells/mm^3^		26	(76.5)	10	(66.7)	16	(84.2)	
High risk of immune activation	38							0.463
No		23	(60.5)	12	(66.7)	11	(55.0)	
Yes		15	(39.5)	6	(33.3)	9	(45.0)	

* Mann–Whitney–Wilcoxon test. ** Either the Khi 2 or Fisher’s exact test. VLLV: very-low-level viremia (defined as viremia < 50 copies/mL detected by clinical assays with quantification cut-offs of <50 copies/mL). High risk of immune activation: at least one among nadir CD4 < 200 cells/mm^3^, AIDS, or VLLV during the follow-up.

**Table 2:** Trajectory of immune activation markers between the inclusion and the end of follow-up.

	**Switch to LA ART (*n* = 18)**					
	**Mean**	**[SD]**	**Median**	**Q1**	**Q3**	***p*** -Value *
sCD14 (ng/mL)						
D0	1.88	[0.71]	1.80	1.46	2.29	0.033
Difference from the end of FU and D0	0.43	[0.77]	0.38	−0.07	2.82	
sCD163 (pg/mL)						
D0	508.04	[278.74]	440.82	313.21	524.49	0.130
Difference from the end of FU and D0	79.87	[334.35]	109.95	−30.76	246.46	
MCP-1 (pg/mL)						
D0	214.92	[66.76]	202.57	165.33	243.21	0.417
Difference from the end of FU and D0	−4.89	[71.93]	−17.97	−40.94	25.29	
IL-6 (pg/mL)						
D0	2.75	[2.27]	1.63	1.50	3.38	0.802
Difference from the end of FU and D0	2.95	[13.33]	0.00	−0.14	0.42	
	**Switch to Oral ART (*n* = 20)**					
	**Mean**	**[SD]**	**Median**	**Q1**	**Q3**	***p*** -Value *
sCD14 (ng/mL)						
D0	3.63	[0.69]	3.55	3.09	3.88	<0.001
Difference from the end of FU and D0	−0.62	[0.50]	−0.50	−0.82	−0.27	
sCD163 (pg/mL)						
D0	371.85	[128.15]	350.49	251.97	477.49	0.093
Difference from the end of FU and D0	44.15	[94.73]	30.86	−40.35	134.13	
MCP-1 (pg/mL)						
D0	70.85	[65.67]	53.67	38.20	73.86	0.114
Difference from the end of FU and D0	−11.31	[40.25]	−9.91	−17.52	0.63	
IL-6 (pg/mL)						
D0	0.85	[2.23]	0.00	0.00	0.00	1.000

* Paired Wilcoxon’s test between M0 and M6. LA: long-acting. D0: day zero M6: month six. FU: follow-up.

**Table 3:** Factors associated with the trajectory of sCD14.

a: Univariate Analysis for sCD14 Absolute Values			
	Trajectory of sCD14 (M6-D0)		
	Spearman’s Rho		*p*-Value
Age	−0.37		0.104
Years of HIV infection	−0.30		0.072
Nadir CD4	−0.14		0.437
Zenith viral load	−0.30		0.179
CD4 at inclusion	0.14		0.406
CD8 at inclusion	0.16		0.336
CD4/CD8 at inclusion	0.07		0.695
Number of ART regimens	−0.10		0.558
Months on the last ART regimen	−0.29		0.076
Years on ART	−0.22		0.197
	Mean	[SD]	*p*-value
Switch to NNRTI			0.004
No	−0.51	[0.76]	
Yes	0.23	[0.79]	
Type of ART when switched			0.001
Injectable	0.43	[0.77]	
Oral	−0.62	[0.58]	
Sex			0.252
Female	0.34	[0.82]	
Male	−0.19	[0.85]	
High blood pressure			0.174
No	0.05	[0.96]	
Yes	−0.38	[0.60]	
Dyslipidemia			0.699
No	−0.07	[0.86]	
Yes	−0.25	[0.87]	
Previous AIDS			0.290
No	−0.12	[0.79]	
Yes	−0.15	[1.31]	
Nadir CD4 < 200, AIDS, or VLLV			0.607
No	−0.12	[0.89]	
Yes	−0.12	[0.81]	
Zenith viral load			0.274
≤100,000 copies/mL	0.15	[0.71]	
>100,000 copies/mL	−0.07	[1.08]	
Contamination route			0.165
Other	−0.31	[0.84]	
MSM	0.06	[0.85]	
b: Multivariate analysis for sCD14 absolute values			
	Adj Coeff	[95% CI]	*p*-value
Years of HIV infection	−0.03	[−0.05; −0.00]	0.032
Months on last ART	0.000	[−0.007; 0.007]	0.967
Switch to NNRTI			0.057
No	ref		
Yes	0.53	[−0.02; 1.08]	
Type of ART			0.039
Injectable	ref		
Oral	−0.60	[−1.17; −0.03]	
Other	ref		
MSM	0.17	[−0.31; 0.65]	
c: Multivariate analysis for sCD14 percent change			
	Adj Coeff	[95% CI]	*p*-value
Years of HIV infection	−0.18	[−3.27; 2.92]	0.909
Months on last ART	−0.21	[−1.08; 0.66]	0.627
Switch to NNRTI			0.290
No	ref		
Yes	−36.05	[−104.25; 32.15]	
Type of ART			0.031
Injectable	ref		
Oral	−74.78	[−142.37; −7.19]	

VLLV: very-low-level viremia. MSM: men who have sex with men. NNRTI: non-nucleoside reverse transcriptase inhibitor.

**Table 4:** Comparison between PWH switching to either oral or LA dual ART according to the risk of immune activation.

	**Switch to LA Dual Therapy**				
	**Risk of Immune Activation**				
	**Low—*n* = 12**		**High—*n* = 6**		
	**Mean**	**[SD]**	**Mean**	**[SD]**	***p*** -Value *
sCD14 D0 (ng/mL)	1.77	[0.70]	2.10	[0.72]	0.482
sCD14 end FU (ng/mL)	2.25	[0.74]	2.45	[1.47]	0.963
Difference sCD14 (end FU—D0)	0.48	[0.64]	0.35	[1.05]	0.437
% sCD14 variation	59.07	[135.24]	12.50	[39.82]	0.553
sCD163 D0 (pg/mL)	494.70	[184.36]	534.72	[433.73]	0.494
sCD163 end FU (pg/mL)	623.28	[333.29]	517.16	[93.91]	0.820
Difference sCD163 (end FU—D0)	128.58	[286.12]	−17.57	[427.66]	0.892
% sCD163 variation	27.18	[58.43]	26.76	[48.75]	0.750
MCP-1 D0 (pg/mL)	206.72	[66.01]	231.31	[71.31]	0.682
MCP-1 end FU (pg/mL)	194.42	[67.81]	241.26	[89.12]	0.335
Difference MCP (end FU—D0)	−12.31	[37.35]	9.96	[118.85]	0.892
% MCP-1 variation	−5.51	[18.91]	12.09	[51.27]	0.820
IL-6 D0 (pg/mL)	1.88	[0.72]	4.50	[3.29]	0.048
IL-6 end FU (pg/mL)	2.58	[1.82]	11.96	[23.07]	0.462
Difference IL-6 (end FU—D0)	0.70	[1.80]	7.45	[23.68]	0.109
% IL-6 variation	39.47	[87.36]	236.97	[657.84]	0.132
	**Switch to Oral Dual ART**				
	**Risk of Immune Activation**				
	**Low—*n* = 11**		**High—*n* = 9**		
	**Mean**	**[SD]**	**Mean**	**[SD]**	***p*** -Value *
sCD14 D0 (ng/mL)	3.69	[0.86]	3.57	[0.43]	0.941
sCD14 end FU (ng/mL)	2.92	[0.47]	3.12	[0.58]	0.425
Difference sCD14 (end FU—D0)	−0.76	[0.71]	−0.44	[0.32]	0.362
% sCD14 variation	−17.92	[15.31]	−12.36	[9.34]	0.456
sCD163 D0 (pg/mL)	344.66	[124.16]	405.09	[132.20]	0.323
sCD163 end FU (pg/mL)	341.21	[120.93]	507.43	[185.91]	0.015
Difference sCD163 (end FU—D0)	−3.45	[69.88]	102.34	[91.02]	0.012
% sCD163 variation	0.53	[24.61]	25.49	[19.25]	0.031
MCP-1 D0 (pg/mL)	78.78	[78.89]	61.17	[47.67]	0.543
MCP-1 end FU (pg/mL)	71.70	[82.07]	44.69	[22.69]	0.470
Difference MCP (end FU—D0)	−7.07	[13.10]	−16.48	[59.82]	1.000
% MCP-1 variation	−11.74	[21.71]	31.61	[153.05]	0.766
IL-6 D0 (pg/mL)	0.38	[1.27]	1.42	[3.02]	0.427
IL-6 end FU (pg/mL)	0.00	[0.00]	4.01	[12.04]	0.315
Difference IL-6 (end FU—D0)	−0.38	[1.27]	2.59	[9.46]	0.669
% IL-6 variation	−8.88	[29.45]	24.41	[114.52]	0.669

* Wilcoxon–Mann–Whitney test. Low risk of immune activation: no nadir CD4 < 200 cells/mm^3^, previous AIDS, or very-low-level viremia during the follow-up. High risk of immune activation: at least one between nadir CD4 < 200 cells/mm^3^, previous AIDS, or very-low-level viremia during the follow-up. ART: antiretroviral treatment. LA: long-acting. FU: follow-up.

## Data Availability

The original contributions presented in the study are included in the article/Supplementary Material; further inquiries can be made to the corresponding author.

## Supplementary Materials

The following supporting information can be downloaded at: https://www.mdpi.com/article/10.3390/pathogens15030316/s1, Figure S1: Trajectories of immune activation markers for each patient from the inclusion to end of follow-up; Table S1: Trajectory of immune activation markers between the inclusion and the end of follow-up, excluding sCD163 and IL-6 outliers; Table S2: Factors associated with i-FABP increase in the Longadapt study; Table S3: Correlations between markers of monocyte–macrophage activation according to the type of switch; Table S4: Trends for immune activation markers, excluding sCD163 and IL-6 outliers, according to risk factors for immune activation.
