# Supplementary material for: Different Trends of Immune Activation Markers When Switching to Either Oral or Injectable Dual Antiretroviral Therapy Based on Integrase Inhibitors in People Living with HIV
Source: Pathogens. 2026 Mar 14;15(3):316. doi: 10.3390/pathogens15030316 (PMC13029144; doi:10.3390/pathogens15030316)
Supplement: Supplementary file 1 [file pathogens-15-00316-s001.zip › Table S2.pdf]

Table S2: Factors associated with i-FABP increase in the Longadapt study

	% I-FABP increase	
	Spearman correlation	p-value
Age	0.23	0.367
Years since known HIV infection (n=16)	0.16	0.529
Nadir CD4 (n=15)	0.32	0.248
Zenith charge virale (n=13)	-0.51	0.074
CD4 at inclusion	-0.18	0.482
CD8 at inclusion	0.42	0.084
CD4/CD8 at inclusion	-0.41	0.091
Antiretroviral regimens received (n=17)	0.17	0.518
Months on last cART before switch	0.35	0.158
sCD14 D0	0.12	0.633
sCD163 D0	0.26	0.290
STNFR1 D0	0.00	1.000
STNFR2 D0	0.04	0.869
Il 6 D0	-0.20	0.419
MCP 1 D0	0.14	0.586
hCRP D0	-0.14	0.586
Total Cholesterol (% increase)	0.18	0.632
HDL Cholesterol (% increase)	0.78	0.012
Triglycerides (% increase)	-0.75	0.018

D0: Day zero
