# Supplementary material for: Different Trends of Immune Activation Markers When Switching to Either Oral or Injectable Dual Antiretroviral Therapy Based on Integrase Inhibitors in People Living with HIV
Source: Pathogens. 2026 Mar 14;15(3):316. doi: 10.3390/pathogens15030316 (PMC13029144; doi:10.3390/pathogens15030316)
Supplement: Supplementary file 1 [file pathogens-15-00316-s001.zip › Table S3.pdf]

Table S3: Correlations between markers of monocyte-macrophage activation according to the type of switch

LA ART

		% increase sCD14	% increase sCD163	sCD14 Trajectory	sCD163 Trajectory
% increase sCD163	Spearman's Rho	-0.54			
	p-value	0.023			
% increase IL-6	Spearman's Rho	0.18	0.02		
	p-value	0.471	0.941		
Trajectory sCD163	Spearman's Rho			-0.57	
	p-value			0.015	
Trajectory IL-6	Spearman's Rho			0.20	0.05
	p-value			0.428	0.832

Oral ART

		% increase sCD14	% increase sCD163	sCD14 Trajectory	sCD163 Trajectory
% increase sCD163	Spearman's Rho	0.24			
	p-value	0.315			
% increase IL-6	Spearman's Rho	0.37	0.19		
	p-value	0.109	0.428		
Trajectory sCD163	Spearman's Rho			0.12	
	p-value			0.607	
Trajectory IL-6	Spearman's Rho			0.33	0.05
	p-value			0.149	0.827
