# Supplementary material for: Different Trends of Immune Activation Markers When Switching to Either Oral or Injectable Dual Antiretroviral Therapy Based on Integrase Inhibitors in People Living with HIV
Source: Pathogens. 2026 Mar 14;15(3):316. doi: 10.3390/pathogens15030316 (PMC13029144; doi:10.3390/pathogens15030316)
Supplement: Supplementary file 1 [file pathogens-15-00316-s001.zip › Table S4.pdf]

Table S4: Trends for immune activation markers excluding sCD163 and IL-6 outliers according to risk factors for immune activation

	Switch to LA dual therapy				
	Risk of immune activation				
	Low - n=12		High - n=6		
	mean	[SD]	mean	[SD]	p-value*
sCD163 D0 (pg/ml)	466.79	[164.64]	359.78	[74.90]	0.221
sCD163 end of FU (pg/ml)	482.71	[248.33]	517.16	[93.91]	0.529
Difference sCD163	22.29	[215.58]	154.61	[79.31]	0.240
% sCD163 increase	7.52	[46.35]	44.58	[24.28]	0.083
IL-6 D0 (pg/ml)	1.88	[0.72]	2.51	[1.07]	0.282
IL-6 end of FU (pg/ml)	1.88	[0.77]	2.55	[1.25]	0.388
Difference IL-6	-0.01	[0.68]	-0.56	[0.84]	0.170
% IL-6 increase	3.91	[26.48]	-17.34	[22.63]	0.170
	**Switch to oral dual ART**				
	**Risk of immune activation**				
	**Low - n=11**		**High - n=9**		
	mean	[SD]	mean	[SD]	p-value*
sCD163 D0 (pg/ml)	344.66	[124.16]	405.09	[132.20]	0.323
sCD163 end of FU (pg/ml)	341.21	[120.93]	507.43	[185.91]	0.015
Difference sCD163	-3.45	[69.88]	102.34	[91.02]	0.012
% sCD163 increase	0.53	[24.61]	25.49	[19.25]	0.031
IL-6 D0 (pg/ml)	0.38	[1.27]	0.53	[1.48]	0.877
IL-6 end of FU (pg/ml)	0.00	[0.00]	0.00	[0.00]	-
Difference IL-6	-0.38	[1.27]	-0.53	[1.48]	0.877
% IL-6 increase	-8.88	[29.45]	-12.21	[34.53]	0.877

*Wilcoxon-Mann-Whitney test
Low risk of immune activation: no nadir CD4 < 200, previous AIDS or very low-level viremia during the follow-up
High risk of immune activation: at least one between nadir CD4 < 200, previous AIDS or very low-level viremia during the follow-up
ART: antiretroviral treatment
LA: Long acting
FU: Follow-up
